# Imported lassa fever in Germany: molecular characterization of a new lassa virus strain.

**DOI:** 10.3201/eid0605.000504

**Published:** 2000

**Authors:** S Günther, P Emmerich, T Laue, O Kühle, M Asper, A Jung, T Grewing, J ter Meulen, H Schmitz

**Affiliations:** Bernhard-Nocht-Institut für Tropenmedizin, Hamburg, Germany.

## Abstract

We describe the isolation and characterization of a new Lassa virus strain imported into Germany by a traveler who had visited Ghana, Côte D'Ivoire, and Burkina Faso. This strain, designated "AV," originated from a region in West Africa where Lassa fever has not been reported. Viral S RNA isolated from the patient's serum was amplified and sequenced. A long-range reverse transcription polymerase chain reaction allowed amplification of the full-length (3.4 kb) S RNA. The coding sequences of strain AV differed from those of all known Lassa prototype strains (Josiah, Nigeria, and LP) by approximately 20%, mainly at third codon positions. Phylogenetically, strain AV appears to be most closely related to strain Josiah from Sierra Leone. Lassa viruses comprise a group of genetically highly diverse strains, which has implications for vaccine development. The new method for full-length S RNA amplification may facilitate identification and molecular analysis of new arenaviruses or arenavirus strains.


### Imported Lassa Fever in Germany: Molecular Characterization of a New Lassa Virus Strain

**Stephan Günther, Petra Emmerich, Thomas Laue, Olaf Kühle, Marcel Asper, Annegret Jung, Thomas Grewing, Jan ter Meulen, and Herbert Schmitz**
Bernhard-Nocht-Institut für Tropenmedizin, Hamburg, Germany

We describe the isolation and characterization of a new Lassa virus strain imported into Germany by a traveler who had visited Ghana, Côte D'Ivoire, and Burkina Faso. This strain, designated "AV," originated from a region in West Africa where Lassa fever has not been reported. Viral S RNA isolated from the patient's serum was amplified and sequenced. A long-range reverse transcription polymerase chain reaction allowed amplification of the full-length (3.4 kb) S RNA. The coding sequences of strain AV differed from those of all known Lassa prototype strains (Josiah, Nigeria, and LP) by approximately 20%, mainly at third codon positions. Phylogenetically, strain AV appears to be most closely related to strain Josiah from Sierra Leone. Lassa viruses comprise a group of genetically highly diverse strains, which has implications for vaccine development. The new method for full-length S RNA amplification may facilitate identification and molecular analysis of new arenaviruses or arenavirus strains.

Transmission of Lassa virus (family *Arenaviridae*) from its natural rodent reservoir to humans can cause hemorrhagic fever, a clinical syndrome associated with high death rates. Lassa fever is endemic in West Africa and has been reported from Sierra Leone, Guinea, Liberia, and Nigeria (1-4). The geographically restricted occurrence of the disease is not well understood as its rodent host (*Mastomys* species) is prevalent in much larger areas of sub-Saharan Africa. The importation of Lassa virus into other regions, for example by travelers, is rare, with only a few cases documented (5-9). Although imported disease often raises public concern because of the possibility of human-to-human transmission; the highly pathogenic nature of the virus; and the lack of an effective, safe therapy, the actual risk for infection from an imported case appears to be low (5,7), and adequate guidelines have been published for disease management in patients and contacts (5,10).

Arenaviruses can be divided phylogenetically, serologically, and geographically into two major complexes: the Old World complex (e.g., Lassa virus, lymphocytic choriomeningitis virus [LCMV]) and the New World complex (e.g., Tacaribe virus, Junin virus, Machupo virus) (11). Isolates of Lassa virus also differ in their genetic, serologic, and pathogenic characteristics (11-13). This variability is evidenced by the poor cross-complement fixation and cross-neutralization among Lassa virus isolates of different geographic origins (3,14). Serologic differences were demonstrated by testing a panel of Lassa virus-specific monoclonal antibodies against many Lassa virus isolates (13).

The single-stranded arenavirus genome consists of a small (S) and a large (L) RNA fragment, sizes 3.4 kb and 7 kb, respectively. The S RNA encodes the viral glycoprotein precursor protein (GPC) and the nucleoprotein (NP). The L RNA encodes the viral polymerase and a small, zinc-binding (Z) protein. Sequencing of the complete S RNA of two Lassa virus strains, originating from Sierra Leone (strain Josiah) (15) and Nigeria (strain Nigeria) (16), as well as sequencing of short S RNA fragments of additional isolates (e.g., strain LP from Nigeria) (11,17,18) showed considerable genetic

Address for correspondence: Stephan Günther, Bernhard-Nocht-Institut für Tropenmedizin, Bernhard-Nocht-Strasse 74, D-20359 Hamburg, Federal Republic of Germany; Fax:

differences. Sequence analysis of the full-length S RNA of a large number of isolates has been complicated by technical problems such as the necessity to produce enough virus in cell culture for direct cloning (15,16) or localization of conserved regions within the S RNA for polymerase chain reaction (PCR) primers (18).

We report the isolation and sequence characterization of a new Lassa virus strain from a traveler who imported the virus into Germany. This novel strain originates from an area of West Africa where Lassa fever has not yet been reported. To facilitate molecular analysis of new Lassa virus isolates, a long-range reverse transcription (RT)-PCR was established. The primers bind to highly conserved RNA termini and allow amplification of full-length S RNA directly from serum.

### Methods

### The Patient

A 23-year-old German woman became ill with fever and flulike symptoms after traveling through three West African countries (Figure 1). In Abidjan, Côte D'Ivoire, she visited the outpatient department of Centre Hospitalier Universitaire de Cocody, where her illness was diagnosed as malaria. She returned to Germany on day 6 of illness and was hospitalized at the Diakonie Hospital at Schwäbisch Hall. After the diagnoses of malaria and bacterial infection were ruled out, the patient was transferred on day 9 of illness to the department of tropical diseases at the Missionsärztliche Klinik in Würzburg, where she was noted to have fever, pharyngitis, diarrhea, and pleural effusion. Lassa fever was suspected, and serum was sent to the Bernhard-Nocht-Institut, Hamburg, where Lassa virus infection was diagnosed by PCR and virus isolation. Despite immediate ribavirin treatment and intensive care, the patient's clinical condition deteriorated, and she died on day 14 of illness with hemorrhage, organ failure, and encephalopathy (19).

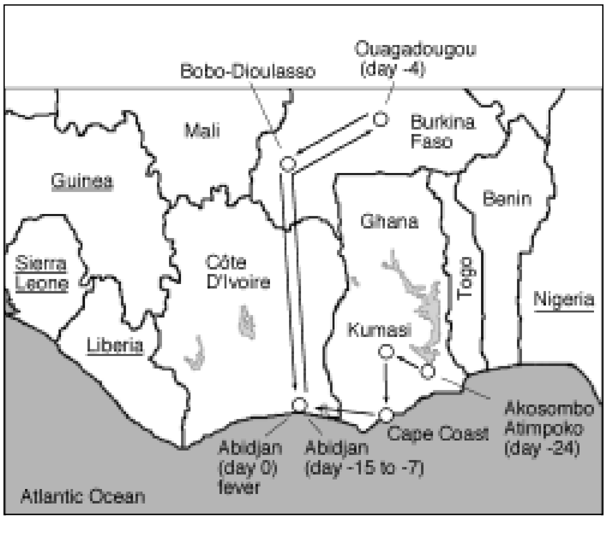

Figure 1. Map of West Africa and travel history of the patient before the onset of febrile illness (day 0). Countries in which Lassa fever is endemic (1-4) are underlined.

### Virus Isolation and Detection by Immunofluorescence and Immunoblot

In the biosafety level 4 facility, Vero cells grown in 10 mL of Leibowitz medium were injected with 1 mL, 0.1 mL, 0.01 mL, and 0.001 mL of serum. The cell culture was examined daily by immunofluorescence for Lassa virus infection as well as morphologic changes. Cells were harvested, spread onto immunofluorescence slides, air-dried, and acetone-fixed. Immunofluorescence was performed by using Lassa virus NP-specific monoclonal antibody L2F1 (20) (dilution of 1:50) and fluorescein isothiocyanate-labeled anti-mouse immunoglobulin (Ig)G diluted 1:60 (Dianova, Hamburg, Germany ).

For immunoblot analysis, cells were harvested and pelleted by centrifugation. The cell pellet was lysed in SDS loading buffer and boiled for 5 min. Total cell lysate was separated in an sodium dodecyl sulfate (SDS)-15% polyacrylamide gel, and proteins were transferred to nitrocellulose membrane (Schleicher & Schuell, Germany). Lassa virus Z protein was detected by chemiluminescence with polyclonal chicken anti-Z serum (dilution 1:5,000) and peroxidase-labeled anti-chicken IgY (dilution 1:2,000) (Dianova) as secondary antibody.

### RT-PCR of S RNA

Virus RNA was isolated from 140 µL of serum or cell culture supernatant of Lassa virus and LCMV-infected Vero and L cells, respectively, by using the QIAamp Viral RNA kit (Qiagen, Hilden, Germany) according to the manufacturer's instructions (elution of RNA in 50 µL of buffer). For reverse transcription of the full-length S RNA, purified RNA (3-6 µL) was incubated with 20 pmol of RT primer (CGCACCGDGGATCCTAG GC) in an 8-µL assay at 70°C for 15 minutes. The mixture was quickly chilled on ice and then centrifuged. A 19-µL reaction premix containing 8 µL RNA-primer mix, 50 mM Tris-HCl (pH 8.3), 75 mM KCl, 3 mM $MgCl_2$, 10 mM DTT, and 500 µM dNTP was incubated at 50°C for 2 minutes. Then 200 units (1 µL) Superscript II reverse transcriptase (Life Technologies, Karlsruhe, Germany) and a drop of mineral oil were added. The reaction was run with the following temperature profile: 50°C for 30 minutes, 55°C for 5 minutes, 50°C for 20 minutes, 60°C for 1 minute, and 50°C for 10 minutes. The enzyme was inactivated at 70°C for 15 minutes. RNA was removed by adding 2 units (1 µL) RNase H (Life Technologies) and incubating at 37°C for 20 minutes. cDNA derived from full-length S RNA was amplified by using the Expand High Fidelity PCR System (Roche Molecular Biochemicals, Mannheim, Germany) with a hot start. A 45-µL reaction premix containing 1 µL of cDNA, 1X reaction buffer with 1.5 mM $MgCl_2$, 200 µM dNTP, and 0.3 µM primers PCR1 to PCR4 in different combinations (PCR1, tatggcgcgcCGCAC CGDGGATCCTAGGC; PCR2, tatggcgcgcCGCAC CGAGGATCCTAGGCATT; PCR3, tatggcgcgcCG CACCGGGGATCCTAGGCAAT; PCR4, tatggcgc gcCGCACCGGGGATCCTAGGCTT; PCR5, tatgg cgcgcCGCACCGDGGATCCTAGGCWWT; heterologous sequences to facilitate cloning via *Asc*I in lower case) was overlaid with 2 drops of oil and heated to 55°C. Subsequently, 5 µl of enzyme mixture containing 2.6 units *Taq* and *Pwo* polymerase in 1X reaction buffer with 1.5 mM $MgCl_2$ was added. PCR was run for 40 cycles with 94° for 1 minute, 55°C for 1.5 minutes, and 72°C for 3 minutes with an increment of 2 minutes after every 10 cycles in a Robocycler (Stratagene, La Jolla, California).

In a separate PCR, a 340-bp fragment of the S RNA was amplified by using Superscript One-Step RT-PCR System (Life Technologies) and primers 36E2 (ACCGGGGATCCTAGGCATTT) and 80F2 (ATATAATGATGACTGTTGTTCTTT GTGCA) as described previously (18).

### Sequence Determination

PCR products were purified by using the QIAquick PCR purification kit (Qiagen) and were directly sequenced with the BigDye Terminator AmpliTaq kit (Applied Biosystems, Weiterstadt, Germany). Extension products were separated on an ABI 377 automated sequencer (Applied Biosystems). The 340-bp fragment was sequenced by using primers 36E2 and 80F2. The full-length S RNA amplification product of independent RT-PCRs was pooled, and plus and minus strands were sequenced by the following primers (numbers denote the position of the 5'-nucleotide of the primer in the genomic sequence of Lassa virus S RNA, strain Josiah; the sequences of LV-$S^J$ and LV-$S^{AV}$ primers are derived from strain Josiah and strain AV, respectively): LV-$S^J$ 1-plus (GCACCGGGGATCCTAGGCATTTTTGGTTGC); LV-$S^J$ 359-plus (GGACTAGAACTGACCTTGACC AACAC); LV-$S^{AV}$ 834-plus (GCACATTCACGTGG ACACTGTCAGA); LV-$S^{AV}$ 1032-plus (TGAAAT CTGAAGCACAAATGAGCAT); LV-$S^{AV}$ 1883-plus (GTGATTCAAGAAGCTTCTTTATGTC); LV-$S^{AV}$ 2372-plus (AGATTTTGTAGAGTATGTTT CATA); LV-$S^J$ 2937-plus (TGCACTTAATGGCCTTTCTG TTCT); LV-$S^{AV}$ 479-minus (GGTGGAAAGTTGA GATTAT GCTCAT); LV-$S^J$ 991-minus (CATGTC ACAAAATTCCTCATCATG); LV-$S^{AV}$ 1906-minus (ACATAAAGAAGCTTCTTGAATCACA); LV-$S^{AV}$ 1955-minus (ATTGAGGCGCTCCCCCGGAATA TGG); LV-$S^J$ 2618-minus (CTAAATATGATTGAC ACCAAGAAAAG); LV-$S^J$ 3092-minus (AATCAA GCGGTCAACAATCTTGTTGA); and LV-$S^J$ 3402-minus (CGCACAGTGGATCCTAGGCTATTGGA TTGC). Each nucleotide position was sequenced by at least two different primers. The overlapping sequences were identical, and no sequence ambiguities were observed.

### Phylogenetic Analysis

Phylogenetic analysis was performed with the NP gene fragment for which the largest set of arenavirus sequence data exists, at position 1724-2349 of the genomic S RNA of Lassa Josiah (11). NP gene sequences were aligned for Lassa AV and the following arenavirus strains (virus, GenBank/EMBL accession number): Tacaribe, M20304; Ippy, U80003; Mopeia AN20410, U80005; Mopeia AN21366, M33879; Mobala 3076, AF012530; Mobala 3051, U80006; Mobala 3099, U80007; Lassa LP, U80004; Lassa Nigeria, X52400; Lassa Josiah, J04324; LCMV Armstrong, M20869; LCMV WE; M22138; and LCMV MaTu-MX, Y16308. Full-length NP and GPC gene sequences were analyzed for Lassa strains Josiah, Nigeria, and AV, and for Mopeia AN21366. Phylogenetic analysis was performed by using programs of the PHYLIP 3.57c package (21). A gap was treated as a single mutation event. Distance matrix calculation and neighbor-joining (NJ) analysis were conducted with the programs DNADIST and NEIGHBOR, and maximum likelihood (ML) analysis was conducted with the DNAML program. Analyses were performed with default settings on a bootstrapped dataset (100 replicates).

### Results

### Origin and Isolation of the New Lassa Virus Strain

The exact geographic origin of the virus and the mode and date of transmission could not be determined. During the incubation period, which can last up to 3 weeks (22), the patient visited Ghana, Côte D'Ivoire, and Burkina Faso (Figure 1). Therefore, the virus originated from one of these West African countries, where Lassa fever has not been reported.

The virus grew rapidly in Vero cells. Fifteen hours after inoculation, few cells were positive, and after 40 hours virtually all cells were infected in cultures injected with 0.1 mL serum as tested by immunofluorescence. In contrast to previous Lassa virus isolations in Vero cells (23), no substantial cytopathic effects were seen. Whether this observation is due to technical variables (such as inoculation dose or culture duration) or reflects a biologic feature of the isolate is unclear. Immunofluorescence with an NP-specific monoclonal antibody showed the speckled, cytoplasmic pattern typical of Lassa virus NP (13,20) (Figure 2A). Isolation of a Lassa virus was further confirmed by immunoblot analysis of strain AV-infected cells using Lassa virus Z protein-specific antibody. An 11-kDa Z protein was demonstrated (Figure 2B) as has recently been detected in Lassa Josiah-infected cells (24).

### RT-PCR for Amplification of Full-Length S RNA

To improve and simplify molecular analysis of new Lassa virus isolates, a protocol for reverse transcription and PCR amplification of the full-length S RNA was established. The RT primer used for reverse transcription binds to the extreme termini of genomic and antigenomic S and L RNA (Figure 3A). These termini are highly conserved among all arenaviruses. Reverse transcription was performed at a baseline temperature of 50°C with short rises to 55°C and 60°C to resolve the stable RNA secondary structure of the intergenic region. A mixture of *Taq* and *Pwo* polymerases, the latter of which has 3'-5'-exonuclease (proofreading) activity, was used in PCR. This enzyme combination can

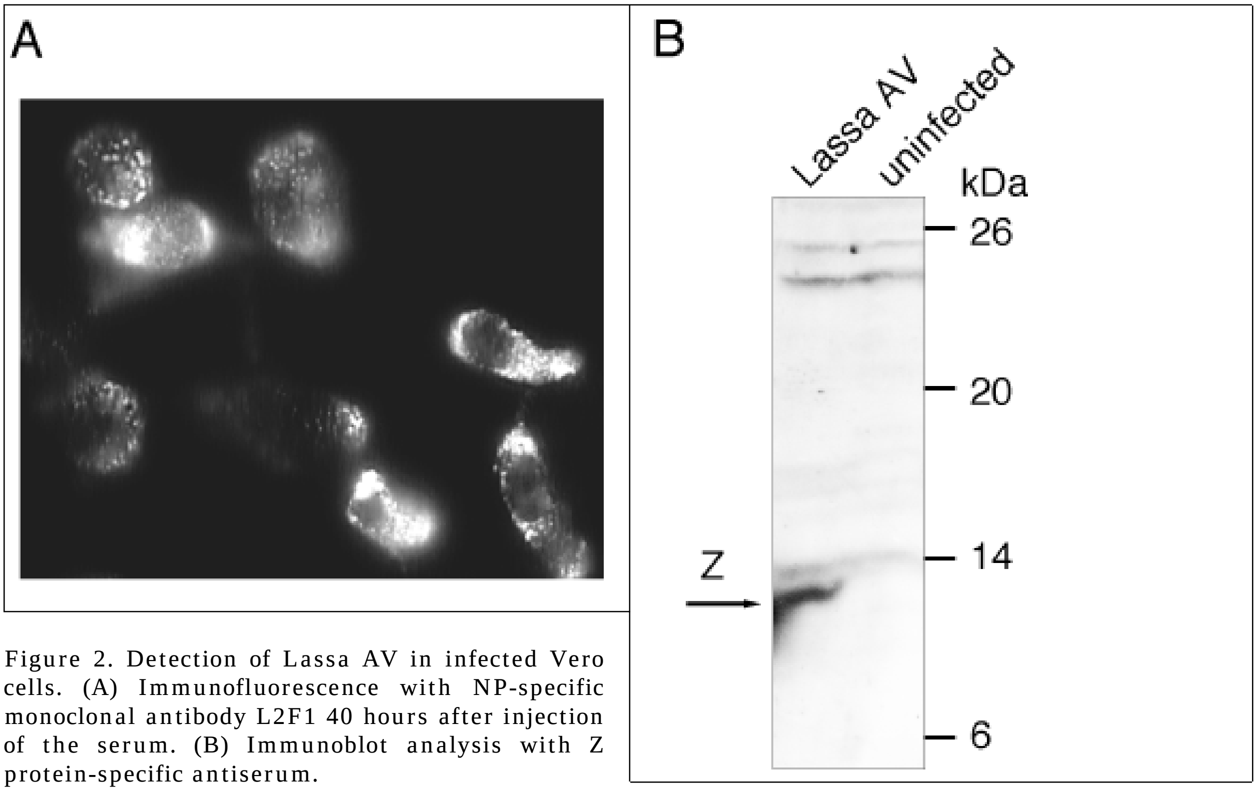

Figure 2. Detection of Lassa AV in infected Vero cells. (A) Immunofluorescence with NP-specific monoclonal antibody L2F1 40 hours after injection of the serum. (B) Immunoblot analysis with Z protein-specific antiserum.

amplify long templates with high fidelity and sensitivity (25). Various primers and primer combinations tested were found suitable for efficient amplification of full-length S RNA of Lassa virus and/or LCMV: PCR1; PCR2; PCR3; PCR2 and PCR3; PCR2, PCR3, and PCR4; and PCR5 (Figure 3B and data not shown). The PCR primers were largely identical to the RT primer but contained heterologous 5'-sequences that allow cloning of the amplification product through the restriction enzyme *Asc*I. The 3'-end of primer PCR1 exactly corresponded to that of the RT primer, but primers PCR2 to PCR5 contained additional two or three nucleotides at their 3'-end. These nucleotides were added to reduce or prevent amplification of shorter products generated by mispriming during reverse transcription. Although the 3'-nucleotides of primers PCR2 and PCR3 did not perfectly fit onto both termini of Lassa virus S RNA, each primer alone was able to amplify the full-length fragment (data not shown). This feature, which may facilitate amplification of strains with mutations in the primer binding site, can be explained by the 3'-5'-exonuclease activity of *Pwo* polymerase, which degrades primers and corrects 3'-mismatches (26). In conclusion, we developed an RT-PCR protocol allowing rapid molecular characterization of S RNA of Lassa virus and LCMV isolates. Because of the high conservation of the primer binding sites, the protocol may also be applied to other arenaviruses.

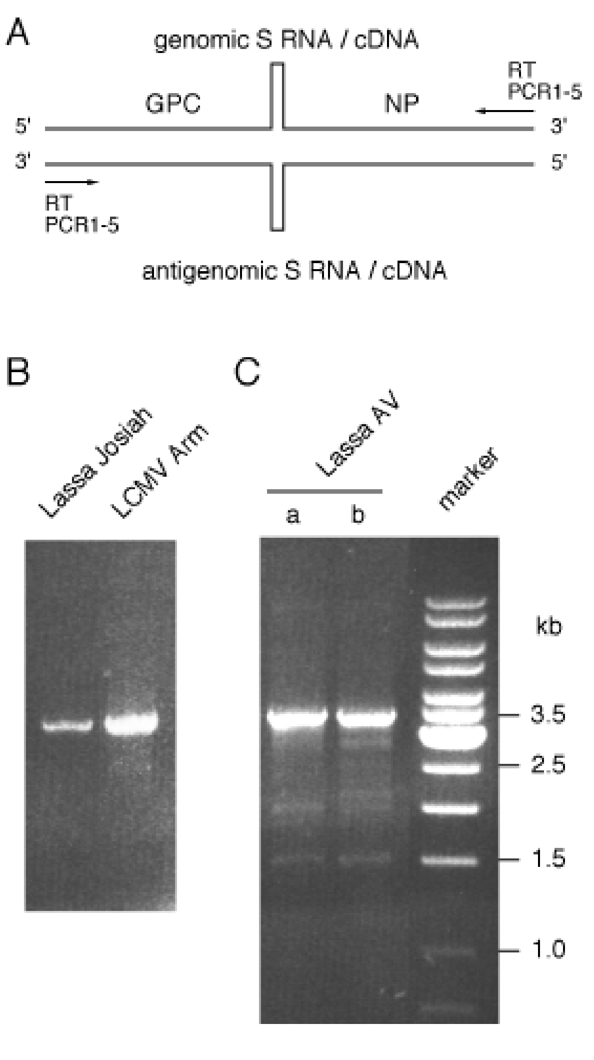

Figure 3. Reverse transcription (RT) and polymerase chain reaction (PCR) amplification of full-length S RNA. (A) Position of the RT and PCR primers at the termini of S RNA. The stem-loop structure in the intergenic region is schematically shown. (B) Amplified S RNA of Lassa Josiah and LCMV Armstrong virus separated in ethidium bromide-stained agarose gel. S RNA was isolated from supernatant of infected cells, and PCR was done with primers PCR2-4. (C) S RNA of Lassa AV was amplified in two RT-PCRs (a and b) and separated in ethidium bromide-stained agarose gel. RNA was isolated from serum, and PCR was performed with primers PCR2-4. Quantification of Lassa virus RNA in the specimen by endpoint titration with the 340-bp PCR assay showed $>10^6$ S RNA molecules/mL serum.

### Sequence Determination of S RNA of Lassa AV and Comparison with Lassa Josiah and Nigeria

S RNA was isolated from the patient's serum and amplified in two RT-PCRs with the primer combination PCR2, PCR3, and PCR4. Both reactions showed a major product at the 3.5-kb position (Figure 3C), indicating that the predominant virus population contained full-length S RNA. Some minor species in the 1.5- to 3-kb size range may represent naturally occurring RNA, with deletion as occasionally seen in arenavirus-infected cell cultures (27), or artifact fragments generated during RT-PCR. The PCR products were purified, pooled, and sequenced. The S RNA sequence was confirmed by sequencing the 340-bp PCR fragment produced by primers 36E2 and 80F2. The overlapping sequences were completely identical. The sequence was sent to GenBank and was assigned accession number AF246121.

Alignment of the S RNA sequence of strain Josiah with that of strains AV and Nigeria showed considerable variability among the three strains (Figure 4). The highest frequency of nucleotide changes, deletions, and insertions was observed at the 3'- and 5'-noncoding regions just upstream of the GPC and NP start codons on the genomic and antigenomic strands, respectively (position 25-55 and 3303-3365). Essentially no

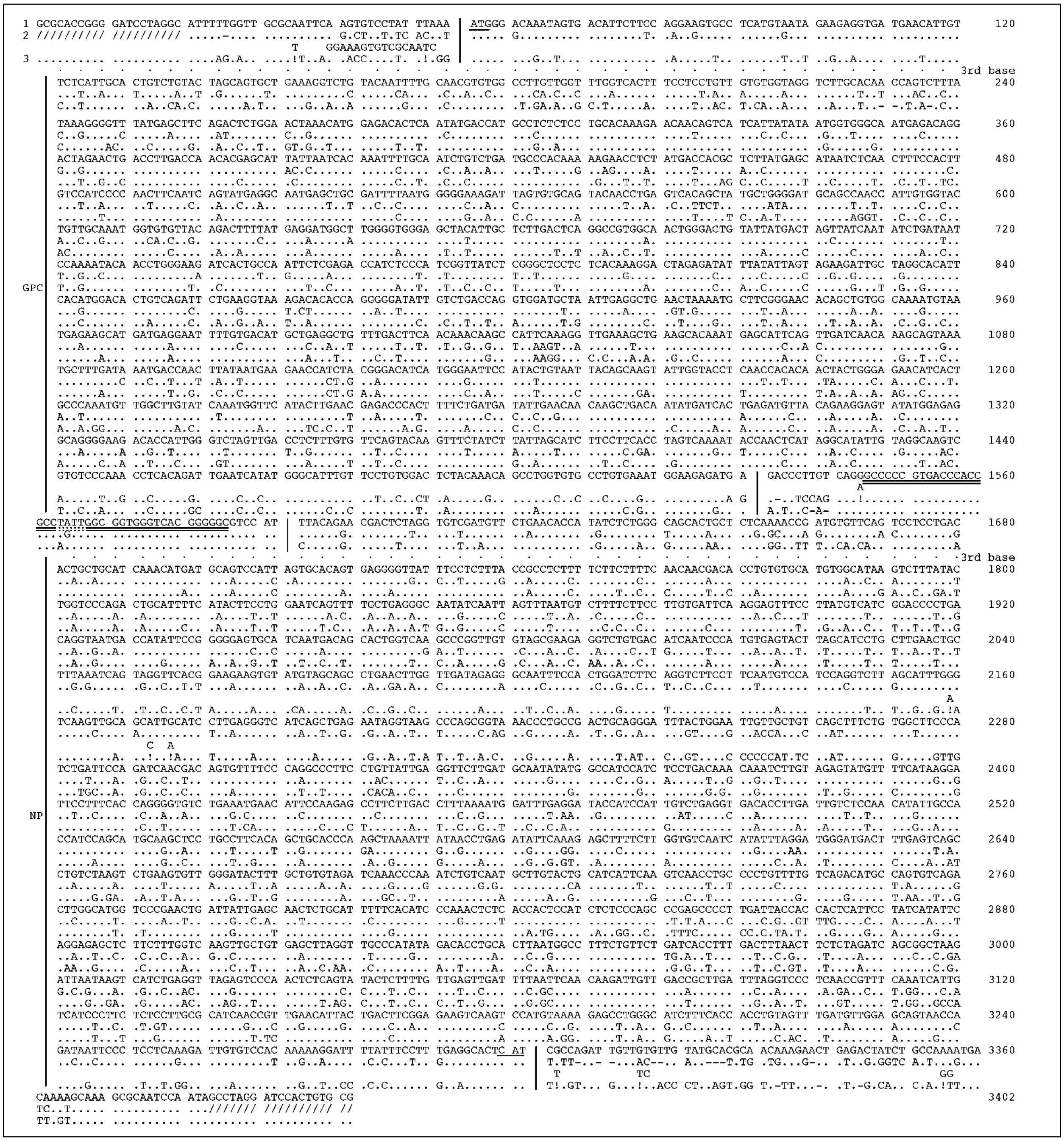

Figure 4. Alignment of the genomic S RNA sequences of Lassa Josiah, AV, and Nigeria (sequences 1, 2, and 3, respectively). The 3'- and 5'-noncoding regions and the intergenic region are separated from the coding regions by vertical bars. Long vertical lines on the left mark the GPC and NP coding regions. Third base positions are marked by a line of dots above each coding region. The GPC and NP start codons are underlined. The stem of the stem-loop structure is underlined by a double line, while the loop is underlined by a dotted line. The RT primer binding sites are indicated by slashes. Inserted nucleotides are shown above the sequence with the position of insertion indicated by a vertical line. The S RNA sequence of strain AV was sent to GenBank and was assigned the accession number AF246121.

nucleotide was conserved in these regions or in a short sequence in the intergenic region between the GPC stop codon and the beginning of the RNA stem-loop structure (position 1532-1540). In contrast to these regions, the RNA stem-loop structure (position 1545-1586) was highly conserved, with no changes in the stem and little variability in the loop. The NP and GPC coding regions differed among the three strains by approximately 20%, almost exclusively because of nucleotide exchanges (Table). The partial NP gene sequence of strain LP differed by 25% from that of strain AV. The mutations were scattered over entire coding regions except for short conserved stretches. The most prominent feature of this variability was the high number of changes at third codon positions, which accounted for approximately 80% of all nucleotide differences (Table). The amino acid variability was considerably lower (5%-9%) than the variability at the nucleotide level (Table). The degree of nucleotide and amino acid sequence divergence was slightly higher in NP than in GPC. Alignment of the GPC amino acid sequences showed differences at the N-terminus and within as well as in the vicinity of the B-cell epitopes (Figure 5) (28,29). The putative GP1/GP2

Table. Nucleotide and amino acid differences between Lassa strains in S RNA coding regions

| Strains[a] | % Nucleotide difference in: GPC | NP | % Changes at 3rd codon positions per total changes in: GPC | NP | % Amino acid difference in: GPC | NP |
|---|---|---|---|---|---|---|
| AV vs. JOS | 16.5 | 19.6 | 84.4 | 81.5 | 5.1 | 6.0 |
| AV vs. NIG | 20.3 | 21.3 | 83.7 | 78.3 | 6.7 | 8.1 |
| NIG vs. JOS | 19.2 | 22.4 | 83.7 | 77.1 | 6.9 | 8.9 |

[a]AV, strain AV; JOS, strain Josiah; NIG, strain Nigeria; GPC = glycoprotein precursor protein; NP = nucleoprotein.

Figure 5. Alignment of the GPC and NP amino acid sequences of Lassa Josiah, AV, and Nigeria (sequences 1, 2, and 3, respectively). B-cell epitopes [GPC 119-133 (28), GPC 124-176 (28), GPC 364-376 (29), NP 123-127 (31)], T-cell epitopes (17), and the putative GPC cleavage site (30) are doubly underlined. Dots above the GPC sequence mark potential N-linked glycosylation sites. Inserted amino acids are shown above the sequence with the position of insertion indicated by a vertical line.

cleavage site (30) was completely conserved, as were potential N-linked glycosylation sites, with the exception of an additional site in Lassa AV and Nigeria at position 272. In NP, two clusters of amino acid variability (position 43-60 and 340-353) were both characterized by a high number of glycine residues at different positions in the three strains (Figure 5). The mapped NP B-cell epitope (31) was conserved, and only a few changes occurred in the T-cell epitopes recently identified in NP (32).

The phylogenetic relationship of Lassa AV to known Old World arenaviruses as well as to the other Lassa strains was analyzed by using partial NP gene sequences. Strain AV segregated with all Lassa strains into a single Lassa group with 100% bootstrap support and was placed in sister relationship with strain Josiah (Figure 6). The latter relationship was confirmed in analyzing the full-length coding regions with 65%/61% (NJ/ML analysis) bootstrap support for the GPC gene and 94%/97% (NJ/ML analysis) bootstrap support for the NP gene.

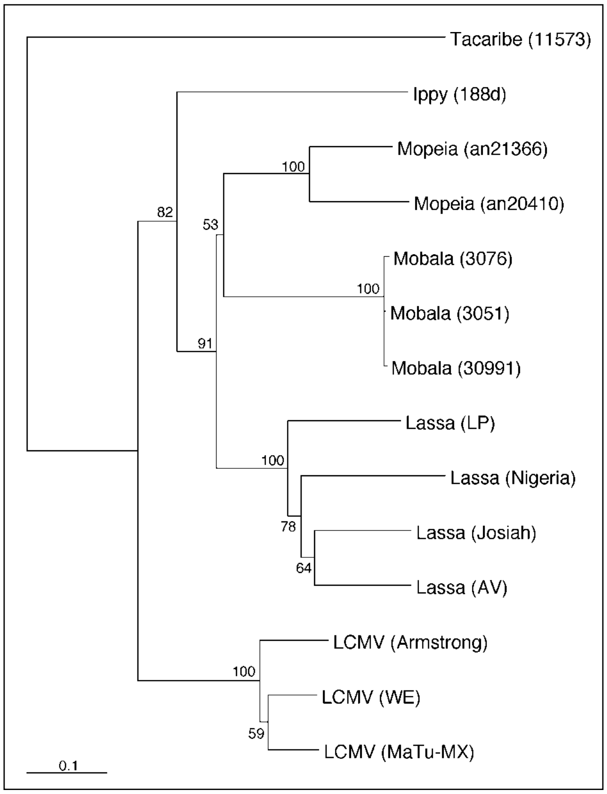

Figure 6. Phylogenetic analysis of Old World arenaviruses, including Lassa AV. The tree was computed for an NP gene fragment by using the neighbor-joining method. Bootstrap support (in %) is indicated at the respective branch. The same topology and similar bootstrap values, except for the terminal lymphocytic choriomeningitis virus branches, were obtained by using the maximum likelihood method. Tacaribe virus belonging to the New World arenaviruses was used as outgroup to root the tree.

### Conclusions

The complete S RNA sequences of three Lassa virus strains—Nigeria (16), Josiah (15), and AV—are now known. All three full-length sequences, as well as the partial S RNA sequence of strain LP (11), differ considerably, suggesting that Lassa viruses comprise a monophyletic yet genetically diverse group. Strain AV appears to be phylogenetically most closely related to strain Josiah. Prominent features of this variability are a high number of substitutions at third-base positions, a high degree of divergence at the 3'- and 5'-noncoding regions just upstream of the NP and GPC start codons, but conservation of the intergenic stem-loop as well as the 19-nucleotide termini, which are conserved among all arenaviruses. Conservation of these termini in strain AV was not directly demonstrated in our study but was suggested by the efficient reverse transcription and amplification with primers binding to these ends. The divergence of the 3'- and 5'-noncoding regions (excluding the conserved termini) indicates either that their function does not depend on a specific primary sequence or that the functional variability of these elements has no major impact on the Lassa virus life cycle. These sequences correspond to the 5'-untranslated regions on the NP and GPC transcripts. Variability in these regions, especially in the so-called KOZAK sequence around the start codon (33), may influence efficiency of translation initiation and, thus, protein expression and virus production. Mutations in noncoding regions may eventually explain pathogenic differences among Lassa virus strains (12), as they have in other viruses (34-36). In contrast to the 3'- and 5'-noncoding regions, the RNA stem-loop structure was highly conserved, suggesting that this element does not allow modification without seriously affecting Lassa virus replication. Of the other arenaviruses, only Mopeia virus has stem-loop sequences in common with Lassa virus (37), which may be one reason that both viruses can form stable reassortants (38). The diverse geographic origins of three of the four prototype strains (LP and Nigeria are both of Nigerian origin) and the relatedness of isolates circulating within an area

(13) suggest geographic clustering of Lassa virus strains. Genetic differences among *Mastomys* species of several regions of West Africa may have led to selection of subspecies-specific Lassa strains. Alternatively, different Lassa strains may have evolved in genetically identical *Mastomys* populations, which are geographically separated because of lack of migration.

The high degree of variability poses problems for the design of diagnostic PCR and sequencing primers. Most of our sequencing primers that were designed on the basis of sequences of strain Josiah and Nigeria failed to anneal to the new strain as a result of several mutations in their binding sites. In addition, the binding site of primer 80F2 (18), which had been designed for diagnostics on the basis of nine Lassa sequences, contained three mutations. As they affected only the 5' half of the primer, performance of the PCR was not seriously reduced, confirming its usefulness for diagnostic purposes. The full-length S RNA RT-PCR may be an alternative for diagnostics because of its highly conserved primer binding sites, although its sensitivity may be somewhat lower.

Phylogenetic analysis showed minor differences in the tree topology of the Old World arenaviruses in comparison to previous analysis (11). In our analysis, Mobala and Mopeia viruses were placed in close relationship, while the previous study indicated that Mobala is most closely related with Lassa virus. However, in both studies, bootstrap support was low and the tree topology depended on the inclusion of changes at the third codon position (11). Placement of Lassa Nigeria, Josiah, and LP differed in both studies in a similar manner. Analysis of additional sequences may be required to elucidate the exact phylogenetic relationship among Mopeia, Mobala, and Lassa viruses, as well as between Lassa virus strains Nigeria, Josiah, and LP.

Development of a vaccine against Lassa virus is a main goal of research (39). Protective immunity is achieved in animals by vaccination with Lassa NP or GPC-expressing vaccinia virus and seems to be mediated by the T-cell response (40-42). However, whether a recombinant vaccine based on a single Lassa protein of a specific strain cross-protects against heterologous Lassa strains has not yet been studied. Recently, several epitopes recognized by Lassa NP-specific CD4+ T-cell clones of one person were mapped (32). Most of them are conserved in at least two of three Lassa strains (Josiah, Nigeria, and AV). The relatively large number of T-cell epitopes recognized, as well as their partial conservation, suggests a level of T-cell cross-reactivity that might be sufficient for cross-protection against heterologous strains after immunization with NP-based vaccines. This view is supported by experiments with Lassa GPC-based vaccines, which indicate CD4+ T-cell-mediated cross-protection even against LCMV (43). Use of the new Lassa virus strain as heterologous challenge virus after immunization with recombinant vaccines, as well as use of its proteins in in-vitro assays to study T-cell cross-reactivity, may enhance our understanding of Lassa virus-specific cross-protective immunity.

### Acknowledgments

We thank Günter Pfaff (Landesgesundheitsamt Baden-Württemberg), Klaus Fleischer (Missionsärztliche Klinik Würzburg), and Hans Peter Geisen (Diakonie Schwäbisch Hall) for providing the patient's clinical data and travel history.

This work was supported by grant E/B31E/M0171/M5916 from the Bundesamt für Wehrtechnik und Beschaffung. The Bernhard-Nocht-Institut is supported by the Bundesministerium für Gesundheit and the Freie und Hansestadt Hamburg.

Dr. Günther is a medical virologist, Department of Virology, Bernhard-Nocht-Institut. His research interest focuses on the genetic variability of arenaviruses and hepatitis B virus.

### References

1. Carey DE, Kemp GE, White HA, Pinneo L, Addy RF, Fom AL, et al. Lassa fever. Epidemiological aspects of the 1970 epidemic, Jos, Nigeria. Trans R Soc Trop Med Hyg 1972;66:402-8.
2. Frame JD, Baldwin JM, Gocke DJ, Troup JM. Lassa fever, a new virus disease of man from West Africa. I. Clinical description and pathological findings. Am J Trop Med Hyg 1970;19:670-6.
3. Monath TP, Maher M, Casals J, Kissling RE, Cacciapuoti A. Lassa fever in the Eastern Province of Sierra Leone, 1970-1972. II. Clinical observations and virological studies on selected hospital cases. Am J Trop Med Hyg 1974;23:1140-9.
4. Monath TP, Mertens PE, Patton R, Moser CR, Baum JJ, Pinneo L, et al. A hospital epidemic of Lassa fever in Zorzor, Liberia, March-April 1972. Am J Trop Med Hyg 1973;22:773-9.
5. Holmes GP, McCormick JB, Trock SC, Chase RA, Lewis SM, Mason CA, et al. Lassa fever in the United States. Investigation of a case and new guidelines for management. N Engl J Med 1990;323:1120-3.

6. Mahdy MS, Chiang W, McLaughlin B, Derksen K, Truxton BH, Neg K. Lassa fever: the first confirmed case imported into Canada. Can Dis Wkly Rep 1989;15:193-8.
7. Zweighaft RM, Fraser DW, Hattwick MA, Winkler WG, Jordan WC, Alter M, et al. Lassa fever: response to an imported case. N Engl J Med 1977;297:803-7.
8. Hirabayashi Y, Oka S, Goto H, Shimada K, Kurata T, Fisher-Hoch SP, et al. An imported case of Lassa fever with late appearance of polyserositis. J Infect Dis 1988;158:872-5.
9. Lloyd G, Barber GN, Clegg JC, Kelly P. Identification of Lassa fever virus infection with recombinant nucleocapsid protein antigen [letter]. Lancet 1989;2:1222.
10. Johnson KM, Monath TP. Imported Lassa fever: reexamining the algorithms. N Engl J Med 1990;323:1139-41.
11. Bowen MD, Peters CJ, Nichol ST. Phylogenetic analysis of the Arenaviridae: patterns of virus evolution and evidence for cospeciation between arenaviruses and their rodent hosts. Mol Phylogenet Evol 1997;8:301-16.
12. Jahrling PB, Frame JD, Smith SB, Monson MH. Endemic Lassa fever in Liberia. III. Characterization of Lassa virus isolates. Trans R Soc Trop Med Hyg 1985;79:374-9.
13. Ruo SL, Mitchell SW, Kiley MP, Roumillat LF, Fisher-Hoch SP, McCormick JB. Antigenic relatedness between arenaviruses defined at the epitope level by monoclonal antibodies. J Gen Virol 1991;72:549-55.
14. Jahrling PB, Frame JD, Rhoderick JB, Monson MH. Endemic Lassa fever in Liberia. IV. Selection of optimally effective plasma for treatment by passive immunization. Trans R Soc Trop Med Hyg 1985;79:380-4.
15. Auperin DD, McCormick JB. Nucleotide sequence of the Lassa virus (Josiah strain) S genome RNA and amino acid sequence comparison of the N and GPC proteins to other arenaviruses. Virology 1989;168:421-5.
16. Clegg JC, Wilson SM, Oram JD. Nucleotide sequence of the S RNA of Lassa virus (Nigerian strain) and comparative analysis of arenavirus gene products. Virus Res 1991;18:151-64.
17. ter Meulen J, Koulemou K, Wittekindt T, Windisch K, Strigl S, Conde S, et al. Detection of Lassa virus antinucleoprotein immunoglobulin G (IgG) and IgM antibodies by a simple recombinant immunoblot assay for field use. J Clin Microbiol 1998;36:3143-8.
18. Demby AH, Chamberlain J, Brown DW, Clegg CS. Early diagnosis of Lassa fever by reverse transcription-PCR. J Clin Microbiol 1994;32:2898-903.
19. World Health Organization. Lassa fever, case imported to Germany. Wkly Epidemiol Rec 2000;75:17-8.
20. Hufert FT, Ludke W, Schmitz H. Epitope mapping of the Lassa virus nucleoprotein using monoclonal anti-nucleocapsid antibodies. Arch Virol 1989;106:201-12.
21. Felsenstein J. PHYLIP (Phylogeny Inference Package) Version 3.57c. [Online] Department of Genetics, University of Washington, Seattle. Available from: URL: http://evolution.genetics.washington.edu/phylip.html [28 January 2000, last date accessed.]
22. McCormick JB, King IJ, Webb PA, Johnson KM, O'Sullivan R, Smith ES, et al. A case-control study of the clinical diagnosis and course of Lassa fever. J Infect Dis 1987;155:445-55.
23. Buckley SM, Casals J. Lassa fever, a new virus disease of man from West Africa. 3. Isolation and characterization of the virus. Am J Trop Med Hyg 1970;19:680-91.
24. Djavani M, Lukashevich IS, Sanchez A, Nichol ST, Salvato MS. Completion of the Lassa fever virus sequence and identification of a RING finger open reading frame at the L RNA 5' end. Virology 1997;235:414-8.
25. Günther S, Sommer G, von Breunig F, Iwanska A, Kalinina T, Sterneck M, et al. Amplification of full-length hepatitis B virus genomes from samples from patients with low levels of viremia: frequency and functional consequences of PCR-introduced mutations. J Clin Microbiol 1998;36:531-8.
26. Roche Molecular Biochemicals. Biochemicals Catalog. Mannheim: Roche Diagnostics GmbH; 2000:p. 50.
27. Francis SJ, Southern PJ. Deleted viral RNAs and lymphocytic choriomeningitis virus persistence in vitro. J Gen Virol 1988;69:1893-902.
28. Krasko AG, Moshnikova AB, Kozhich AT, Tchikin LD, Ivanov VT, Vladyko AS, et al. Lassa virus glycoproteins: antigenic and immunogenic properties of synthetic peptides to GP1. Arch Virol 1990;115:133-7.
29. Weber EL, Buchmeier MJ. Fine mapping of a peptide sequence containing an antigenic site conserved among arenaviruses. Virology 1988;164:30-8.
30. Buchmeier MJ, Southern PJ, Parekh BS, Wooddell MK, Oldstone MB. Site-specific antibodies define a cleavage site conserved among arenavirus GP-C glycoproteins. J Virol 1987;61:982-5.
31. Vladyko AS, Bystrova SI, Malakhova IV, Kunitskaia L, Lemeshko NN, Krasko AG, et al. The localization and preliminary characteristics of antigenic sites (B epitopes) in the nucleocapsid protein of the Lassa virus. Vopr Virusol 1993;38:30-4.
32. ter Meulen J, Badusche M, Kuhnt K, Doetze A, Satoguina J, Marti T, et al. Characterization of human CD4+ T-cell clones recognizing conserved and variable epitopes of the Lassa virus nucleoprotein. J Virol 2000;74:2186-92.
33. Kozak M. Point mutations define a sequence flanking the AUG initiator codon that modulates translation by eukaryotic ribosomes. Cell 1986;44:283-92.
34. Evans DM, Dunn G, Minor PD, Schild GC, Cann AJ, Stanway G, et al. Increased neurovirulence associated with a single nucleotide change in a noncoding region of the Sabin type 3 poliovaccine genome. Nature 1985;314:548-50.
35. Kobiler D, Rice CM, Brodie C, Shahar A, Dubuisson J, Halevy M, et al. A single nucleotide change in the 5' noncoding region of Sindbis virus confers neurovirulence in rats. J Virol 1999;73:10440-6.
36. Spotts DR, Reich RM, Kalkhan MA, Kinney RM, Roehrig JT. Resistance to alpha/beta interferons correlates with the epizootic and virulence potential of Venezuelan equine encephalitis viruses and is determined by the 5' noncoding region and glycoproteins. J Virol 1998;72:10286-91.

37. Wilson SM, Clegg JC. Sequence analysis of the S RNA of the African arenavirus Mopeia: an unusual secondary structure feature in the intergenic region. Virology 1991;180:543-52.
38. Lukashevich IS. Generation of reassortants between African arenaviruses. Virology 1992;188:600-5.
39. ter Meulen J. Lassa fever: implications of T-cell immunity for vaccine development. J Biotechnol 1999;73:207-12.
40. Fisher-Hoch SP, McCormick JB, Auperin D, Brown BG, Castor M, Perez G, et al. Protection of rhesus monkeys from fatal Lassa fever by vaccination with a recombinant vaccinia virus containing the Lassa virus glycoprotein gene. Proc Natl Acad Sci U S A 1989;86:317-21.
41. Clegg JC, Lloyd G. Vaccinia recombinant expressing Lassa-virus internal nucleocapsid protein protects guineapigs against Lassa fever. Lancet 1987;2:186-8.
42. Morrison HG, Bauer SP, Lange JV, Esposito JJ, McCormick JB, Auperin DD. Protection of guinea pigs from Lassa fever by vaccinia virus recombinants expressing the nucleoprotein or the envelope glycoproteins of Lassa virus. Virology 1989;171:179-88.
43. La Posta VJ, Auperin DD, Kamin-Lewis R, Cole GA. Cross-protection against lymphocytic choriomeningitis virus mediated by a CD4+ T-cell clone specific for an envelope glycoprotein epitope of Lassa virus. J Virol 1993;67:3497-506.
